# First evidence of the presence and activity of archaeal C3 group members in an Atlantic intertidal mudflat

**DOI:** 10.1038/s41598-018-30222-1

**Published:** 2018-08-07

**Authors:** Céline Lavergne, Mylène Hugoni, Christine Dupuy, Hélène Agogué

**Affiliations:** 1 Université de La Rochelle – CNRS, UMR 7266, LIENSs, 2 rue Olympe de Gouges, 17000 La Rochelle, France; 2 Escuela de Ingeniería Bioquímica, Pontificia Universidad Católica Valparaíso, Avenida Brasil 2085, Valparaíso, Chile; 3 Université Lyon 1 – UMR CNRS 5557/INRA 1418, Ecologie Microbienne, Villeurbanne, France

## Abstract

The phylogenetic assignment of archaeal communities is constantly evolving, and the recent discovery of new phyla that grouped into superphyla has provided novel insights into archaeal ecology and evolution in ecosystems. In intertidal sediments, archaea are known to be involved in key functional processes such as organic matter turnover, but the ecological relevance of the rarest archaeal groups is poorly investigated, due partly to the lack of cultivated members. The high resolution of microbial diversity provided by high-throughput sequencing technologies now allows the rare biosphere to be described. In this work, we focused on the archaeal C3 group, showing that this phylum is not only present (at the DNA level) independently of sediment depth but also active (at the RNA level) in specific sediment niches depending on vertical physicochemical gradients. Moreover, we highlight the ambiguous phylogenetic affiliation of this group, indicating the need of further research to get new insights into the role of the C3 group.

## Introduction

Recent developments in metagenomic approaches have led to a clear demonstration of the existence of a rare biosphere, defined as low-abundance taxa present in ecosystems (accounting for 0.1 to 0.001% of sequence abundance depending on the study)^1,2^. While previous works had focused on eukaryota^3,4^ and bacteria^5,6^, only a few studies have considered the rare archaeal biosphere^7,8^. However, recent work has demonstrated the existence of different fractions among the rare archaeal biosphere. Some of these rare archaea are taxonomically close to abundant members, and some are always active, while others are inactive, which might represent dormant microorganisms that are able to react to seasonal fluctuations and serve as a local seed bank. A third fraction consists of rare archaea that are uncommon in public databases, inactive, and alien to the studied ecosystem, representing a non local seed bank of potential colonizers^7^. These rare taxa could therefore represent a gene reservoir and might play key roles in biogeochemical cycles^7,9^.

In intertidal mudflats, sharp vertical gradients in salinity, oxidative state and nutrients^10^ shape microbial establishment (at the DNA level)^11^ as well as microbial activity (at the RNA level)^12^. While archaea represent a small fraction of the active prokaryotic community compared with their bacterial counterparts, vertical shifts in major archaeal phyla have been recorded^12^. The taxonomic affiliation of several of these rare archaeal taxa that have been newly discovered in many environmental samples is still under active debate^13,14^. In the present work, we demonstrated that the potentially active archaeal fraction retrieved from coastal sediments was enriched in a classically non-abundant phylum representing an emergent class of *Bathyarchaeota*. These findings raise many questions about the physiology of this group and their adaptation to this peculiar environment.

We were interested in the ecological relevance of the archaeal C3 group, which is still poorly described, as none of its members have yet been cultivated. This group was formerly referred to as subclass MCG-15 of *Bathyarchaeota* (previously known as the *Miscellaneous Crenarchaeotic Group*, MCG)^15^ and has been reported to be widespread in anoxic sediments^16^, hydrothermal vents^17^, and lakes^18^.

## Material and Methods

### Sampling and nucleic acid extraction

Marennes-Oléron Bay is located in the center of the French Atlantic coast. Sampling was performed along ridges at low tide. Triplicate 15-cm-diameter cores were sliced into five layers from 0 to 10 cm below the sediment surface (bsf) then homogenized and stored at −20 °C. DNA was extracted from ~1 g of thawed sediment using the UltraClean Soil kit^®^ (MOBIO, USA) according to the manufacturer’s recommendations to maximize yields.

As described in detail in a previous study^12^, RNA extracts were obtained from five sediment layers of a tidal mesocosm using the Power Soil^TM^ Total RNA Isolation Kit (MOBIO, USA). cDNAs were generated using SuperScript III and random hexamers (Invitrogen, Life Technologies, USA).

### High-throughput sequencing

In the current study, three sets of metabarcoding data were used. First, a set of 16S rRNA genes (DNA level) and a set of 16S rRNA transcripts (RNA level), sequenced via 454 pyrosequencing, as partially described in a previous study^12^, were used to determine the distribution of archaeal taxa in relation to sediment depth. The 454 pyrosequencing procedure is fully described in previous work^12^. Second, to confirm the enrichment of archaeal C3 group sequences in specific niches and obtain longer and more robust sequences for phylogenetic analysis, we re-sequenced the two enriched samples (from 5 to 10 cm below sediment surface) using the Illumina HiSeq platform. Briefly, the Illumina HiSeq approach consisted of amplification of the V3–V5 region of the archaeal 16S rRNA genes using the 519F and 915R primers^18^. High-throughput sequencing was achieved after a multiplexing step using HiSeq Rapid Run 300 bp PE technology in an Illumina HiSeq2500 system (GATC Biotech, Konstanz, Germany). Archaeal 16S rRNA paired-end reads were merged with a maximum of 10% mismatches in the overlap region using FLASh^19^. Denoising procedures consisted of discarding reads outside of the expected length range (*i*.*e*., expected size between 370 and 580 bp) and reads containing ambiguous bases (N). After dereplication, the sequences were clustered into operational taxonomic units (OTUs) using SWARM^20^ with a local clustering threshold. In the present work, the aggregation distance was equal to 3. Chimeras were then removed using VSEARCH^21^, and low-abundance sequences were filtered at 0.005% (*i*.*e*., OTUs with at least 0.005% of all sequences were retained^22^), discarding singletons from the datasets. Taxonomic affiliation was evaluated using both RDP Classifier^23^ and BLASTN+^24^ against the 119 SILVA database^25^. This procedure was automated in the FROGS pipeline^20^. Raw data are available under Sequence Read Archive (SRA) format within the BioProject PRJNA477428.

### Phylogenetic analysis

From the high-throughput sequencing step using the Illumina HiSeq platform, 82 archaeal 16S rRNA gene sequences associated with the C3 group were obtained. All the sequences were analyzed using the ARB software package^26^ (version July 2014) and the corresponding SILVA SSURef 99 database^27^, version 128, released on 07.09.2016. All the sequences were automatically aligned using the SINA tool online^28^. The aligned sequences were added to the tree of the SILVA database. Tree reconstruction was first performed with 241 full 16S rRNA gene sequences using two methods: neighbor joining (NJ, ARB) and maximum likelihood (ML, RAxML^29^ v8.0.24 using the CIPRES gateway^30^) to analyze the topology of the tree. Only the 25 most abundant OTUs (Supplementary Information, Table S1) were selected for the final tree. The final tree was computed with 129 sequences (bacteria *Tenericutes* as the outgroup, the Ancient archaeal group, *Lokiarchaeota*, *Thorarchaeota*, *Thaumarchaeota* Marine Group I, *Bathyarchaeota* and the C3 group) using the neighbor-joining method (Jukes Cantor distance, bootstrapping: 1000 replications) and applying the archaeal positional variability by parsimony (PVP) filter to partial sequences (423 positions). For clarity, only selected subsets of the sequences used to build the tree are shown in the figure.

## Results and Discussion

*Bathyarchaeota* is a recently identified phylum composed of 17 subclasses with a diverse metabolic spectrum, mainly reported as anaerobic^31^. In the last two years, two partial genomes have been obtained for the C3 group (MCG-15 subclass): E09^32^ and B23^33^. These findings agreed with the capacity of the C3 group to fix inorganic CO_2_ via the reductive acetyl-CoA (Wood–Ljungdahl, WL) pathway^33^. The C3 group is the first archaeal taxa capable of homoacetogenesis^33^, as its members use H_2_ as an electron donor to form acetate autotrophically^31^ via a more energy-efficient pathway than bacteria. This lower efficiency of bacteria is due to their requirement for ATP to generate formate^33^. Thus, these archaea appear to be better adapted to low-energy environments^33^. Although previous work indicated that members of *Bathyarchaeota* are also implicated in the methane cycle and could carry out methanogenesis^34^, only subclass MCG-8 appears to exhibit this peculiarity thus far. The reported partial genomes for the C3 group did not contain a methyl coenzyme M reductase gene (*mcrA* gene) related to methanogenesis. Recent findings suggest that all *Bathyarchaeota* may have been methanogens in the past but that most of them appear to have lost this capacity^35^. The authors of this study also suggest that subclass MCG-8 may be the first methanogens to lose methanogenesis. The metabolic diversity of *Bathyarchaeota* makes it difficult to appreciate the ecological role of each *Bathyarchaeota* subclass, and more environmental records and information are needed to better understand one of the most important benthic archaeal phyla.

**Figure 1:**
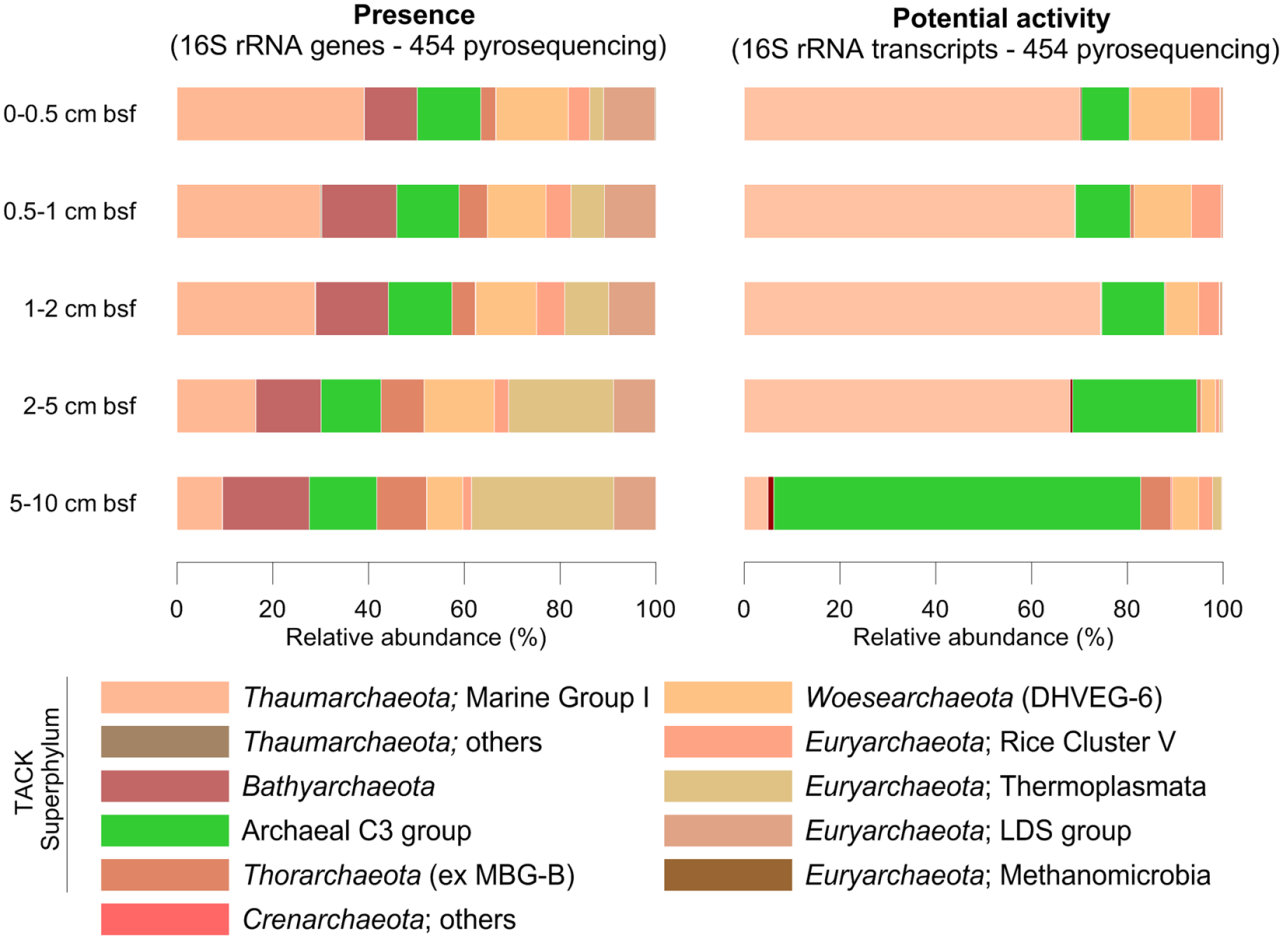
Relative abundance of archaeal 16S rRNA genes and transcripts in coastal sediments. The left panel shows the relative abundance of archaeal 16S rRNA genes recovered *in situ* on the 5^th^ of July 2012 in an intertidal mudflat of Marennes-Oléron Bay. The right panel shows the relative abundance of archaeal 16S rRNA transcripts recovered in a tidal mesocosm using sediments from the same Marennes-Oléron mudflat. The relative abundance of the 16S rRNA genes or transcripts and the affiliation of the total archaeal OTUs are presented at the class level among the five layers below the sediment surface (bsf).

In the current study, we demonstrated the ubiquity of the archaeal C3 group at different vertical sediment depths in an Atlantic intertidal mudflat through an *in situ* study conducted at the DNA level. The abundance of this group was evaluated along a vertical gradient from 0 to 10 cm below sediment surface (bsf), and this abundance was stable throughout the sediment cores, comprising up to 13% of the obtained 16S rRNA archaeal gene sequences (Fig. 1). Despite the stability in terms of 16S rRNA gene sequence numbers at different sediment depths, a mesocosm experiment corresponding to the same sampling sites and depths clearly demonstrated for the first time that this rare group is highly active in mudflat sediments in particular (at the RNA level, Fig. 1). Indeed, the diversity of archaeal 16S rRNA transcripts showed that a considerable proportion of archaeal C3 group sequences varied greatly according to depth and associated vertical gradients (*i*.*e*., 13% of total archaeal 16S rRNA transcript sequences were found from 2 to 5 cm bsf, and up to 70% were in the deeper zone, from 5 to 10 cm bsf; Fig. 1). These findings suggest the presence of ecological niches that favor the activity of these archaea, although they remain rare along the vertical gradient. These findings were possible because of the use of archaeal 16S rRNA transcript sequencing, whereas most other related studies have described only microbial diversity at the 16S rRNA gene level or have lacked this specific focus on archaeal communities, thus, potentially overlooking active communities and archaeal diversity.

**Figure 2:**
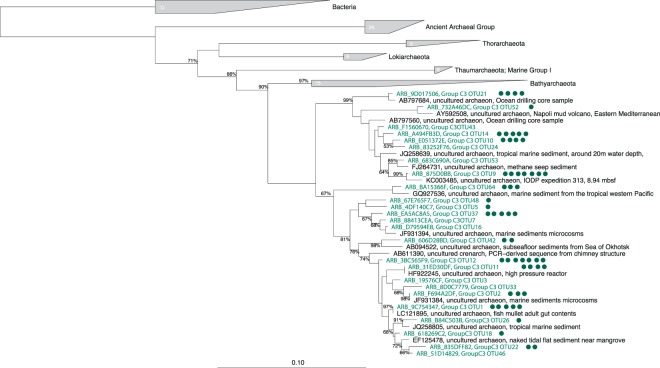
Phylogenetic tree constructed with the most abundant C3 group-affiliated OTUs (representing 82 sequences) retrieved in our study. Sequences from the current study are highlighted in green in the tree and the circles indicated the number of OTUs they represent. The tree was built using neighbor-joining calculations in ARB at 423 positions and applying PVP filtering and 1000 bootstraps. Bootstrap values >50% are displayed.

While the C3 group is considered a class of *Bathyarchaeota*^15^, the SILVA database considers C3 as an emergent group that is more closely related to *Thaumarchaeota*. To assess the phylogenetic position of the C3 group transcripts retrieved in the present work, we re-sequenced the two enriched samples previously identified through 454 pyrosequencing (from 5 to 10 cm bsf) using the Illumina HiSeq 2 * 300 bp platform to increase sequencing depth and quality. The phylogenetic analysis (Fig. 2) of the 82 sequences of C3 group-affiliated 16S rRNA transcripts retrieved from Marennes-Oléron bay confirmed that the C3 group is closely related to *Bathyarchaeota*.

This phylogenetic analysis separated the C3 OTUs obtained in the current study into two different groups. The first contained environmental sequences from the subseafloor, shallow sediment and extreme environments such as the Napoli mud volcano, while the second group might correspond to a coastal group. Interestingly, 16 OTUs were placed in the tree near benthic environmental sequences originating in the Aber-Benoît tidal basin, which is located in a neighboring region of the current study site^36^.

The relationship of the archaeal C3 group with both *Thaumarchaeota* and *Bathyarchaeota* as a part of the TACK superphylum is clear, but whether the C3 group is a class of *Bathyarchaeota* cannot be determined using only the V3–V4 region of the 16S rRNA gene marker. A metagenomic and metatranscriptomic survey in C3 group-enriched ecosystems would help to reveal the metabolic functions of this mysterious, non-abundant archaeal group. Thus, we highlight the necessity of (1) exploring potentially active biodiversity at the RNA level to detect key ecological players among nonabundant taxa; and (2) studying further the C3 group, as it could be a key taxon for better understanding biogeochemical processes in coastal sediments.

## Electronic supplementary material

Supplementary Information
