# Supplementary material for: First evidence of the presence and activity of archaeal C3 group members in an Atlantic intertidal mudflat
Source: Sci Rep. 2018 Aug 7;8:11790. doi: 10.1038/s41598-018-30222-1 (PMC6081377; doi:10.1038/s41598-018-30222-1)
Supplement: Supplementary file 1 — Supplementary Information [file 41598_2018_30222_MOESM1_ESM.docx]

**First evidence of the presence and activity of archaeal C3 group members in an Atlantic intertidal mudflat**

C. Lavergne ^1,2§^, M. Hugoni ^3§^, C. Dupuy ^1^, H. Agogué ^1^

^1^ Université de La Rochelle – CNRS, UMR 7266, LIENSs, 2 rue Olympe de Gouges, 17000 La Rochelle, France

^2^ Escuela de Ingeniería Bioquímica, Pontificia Universidad Católica Valparaíso, Avenida Brasil 2085, Valparaíso, Chile

^3^ Université Lyon 1 – UMR CNRS 5557 / INRA 1418, Ecologie Microbienne, Villeurbanne, France

^§^ C. Lavergne and M. Hugoni contributed equally to this work.

All datasets described in the paper will be publicly available in the SRA deposit in NCBI un the BioProject PRJNA477428.

**Table S1.** Number of sequences and relative abundance of each OTU affiliated to C3-group in this study

|  | Number of C3-affiliated sequences | |  | Relative abundance (% sequences in the sample) | |
| --- | --- | --- | --- | --- | --- |
|  | Day | Night |  | Day | Night |
| OTU 1 | 69705 | 72337 |  | 25.8% | 26.8% |
| OTU 2 | 35731 | 30920 |  | 13.2% | 11.4% |
| OTU 3 | 21382 | 22356 |  | 7.9% | 8.3% |
| OTU 5 | 16367 | 17674 |  | 6.1% | 6.5% |
| OTU 7 | 18455 | 18388 |  | 6.8% | 6.8% |
| OTU 9 | 10225 | 7749 |  | 3.8% | 2.9% |
| OTU 10 | 9408 | 10508 |  | 3.5% | 3.9% |
| OTU 11 | 7682 | 7943 |  | 2.8% | 2.9% |
| OTU 12 | 7729 | 7025 |  | 2.9% | 2.6% |
| OTU 14 | 4696 | 5395 |  | 1.7% | 2.0% |
| OTU 16 | 4980 | 4833 |  | 1.8% | 1.8% |
| OTU 18 | 5816 | 4446 |  | 2.2% | 1.6% |
| OTU 21 | 4412 | 4125 |  | 1.6% | 1.5% |
| OTU 22 | 7682 | 5881 |  | 2.8% | 2.2% |
| OTU 24 | 4797 | 4220 |  | 1.8% | 1.6% |
| OTU 26 | 3838 | 3108 |  | 1.4% | 1.2% |
| OTU 33 | 2551 | 2438 |  | 0.9% | 0.9% |
| OTU 37 | 2628 | 1723 |  | 1.0% | 0.6% |
| OTU 42 | 1951 | 2041 |  | 0.7% | 0.8% |
| OTU 43 | 2180 | 1892 |  | 0.8% | 0.7% |
| OTU 46 | 2020 | 1576 |  | 0.7% | 0.6% |
| OTU 48 | 1842 | 2147 |  | 0.7% | 0.8% |
| OTU 52 | 1702 | 1342 |  | 0.6% | 0.5% |
| OTU 53 | 2194 | 1104 |  | 0.8% | 0.4% |
| OTU 64 | 1109 | 1677 |  | 0.4% | 0.6% |
